# Supplementary material for: Sexually dimorphic effects of prenatal alcohol exposure on the murine skeleton
Source: Biol Sex Differ. 2024 Jun 18;15:51. doi: 10.1186/s13293-024-00626-y (PMC11186175; doi:10.1186/s13293-024-00626-y)
Supplement: Supplementary file 1 — Supplementary Material 1 [file 13293_2024_626_MOESM1_ESM.docx]

**Supplementary Information:** *Sexually dimorphic effects of prenatal alcohol exposure on the murine skeleton*

Lucie E Bourne, Soher N Jayash, Lysanne V Michels, Mark Hopkinson, Fergus M Guppy, Claire E Clarkin, Paul Gard, Nigel Brissett, Katherine A Staines

**Suppl. Figure 1. Additional trabecular parameters in 12-week-old mice.** µCT analysis of trabecular bone parameters, including (**A**) tissue surface, (**B**) bone surface to bone volume ratio, (**C**) trabecular thickness, (**D**) trabecular separation, (**E**) degree of anisotropy, (**F**) bone mineral density (BMD), and (**G**) bone surface to tissue volume in PAE and control males and females. Data are presented as mean ± SEM with points showing individual animals. *= *p*<0.05, **= *p*<0.01, ***= *p*<0.001, ****= *p*<0.0001.


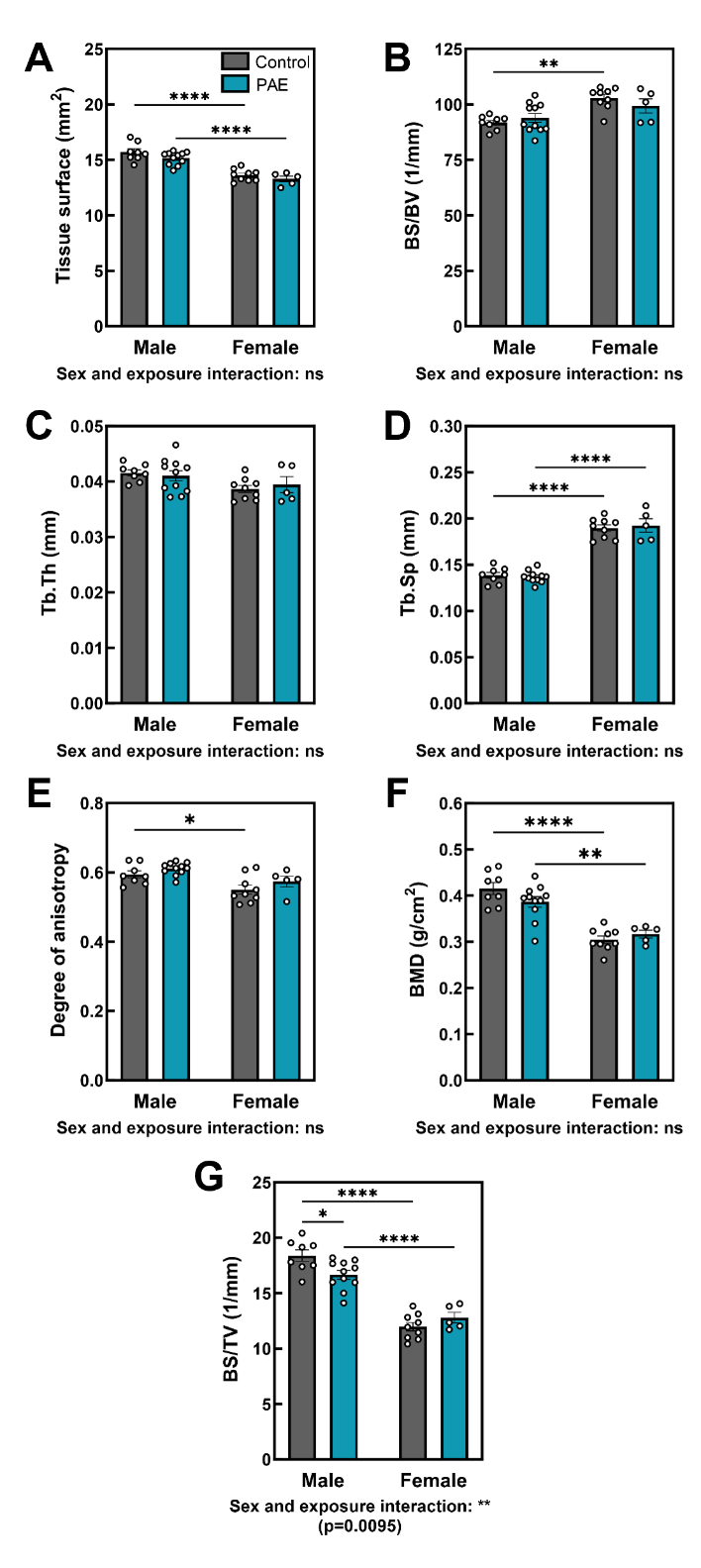


**Suppl. Figure 2. Additional trabecular bone parameters and growth plate bridges in 4-week-old mice.** µCT analysis of trabecular bone parameters, including (**A**) intersection surface, (**B**) tissue surface, (**C**) bone surface to bone volume ratio, (**D**) bone surface to tissue volume ratio, (**E**) trabecular thickness, (**F**) trabecular separation, (**G**) degree of anisotropy, and (**H**) bone mineral density (BMD) in control and PAE males and females. (**I**) Number of growth plate bridges in control and PAE mice of both sexes. Data are presented as mean ± SEM with points showing individual animals. *= *p*<0.05, **= *p*<0.01, ***= *p*<0.001, ****= *p*<0.0001.


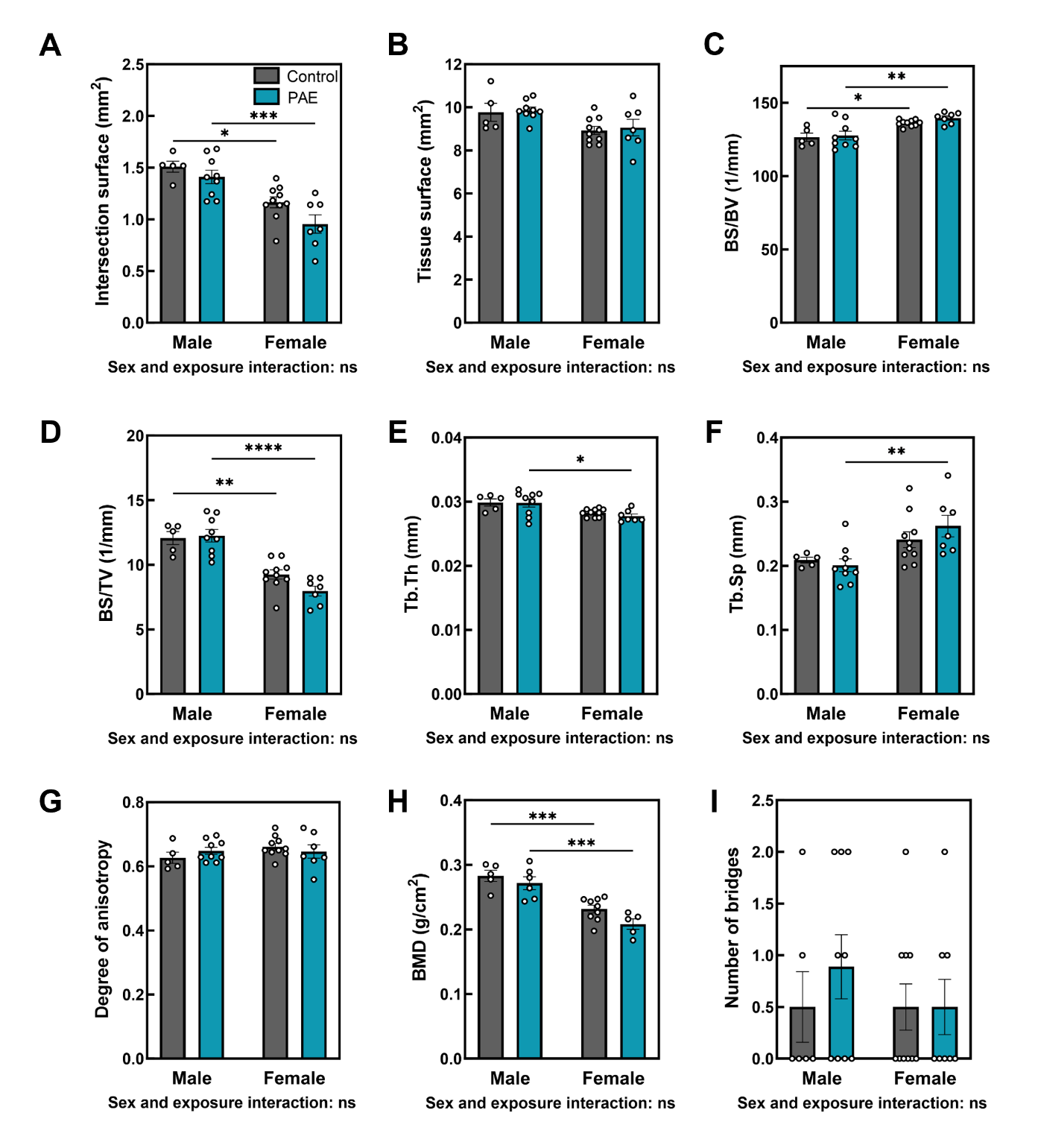


**Suppl. Table 1: Litter size and sex distribution of control and PAE offspring.** *Data represented as mean ± SEM*

|  | **Litter size** | **Ratio of males to females** |
| --- | --- | --- |
| **Control** | 6.75 ± 0.70 | 0.58 ± 0.07 |
| **PAE** | 7.18 ± 0.71 | 0.56 ± 0.04 |

**Suppl. Table 2: Primer sequences or catalogue numbers (QIAGEN QuantiTect primer assay) used for RT-qPCR.**

| **Gene** | **Sequence or catalogue number** |
| --- | --- |
| *β-actin* | Forward: *gatgtggcaccacaccttct*  Reverse: *ggggtgttgaaggtctcaaa* |
| *Ashg* | QT00142030 |
| *Bglap* | Forward: *ccgggagcagtgtgagctta*  Reverse: *tagatgcgtttgtaggcggtc* |
| *Bmp6* | QT00172627 |
| *Flt1* | QT00096292 |
| *Tgfbr1* | QT00135828 |
| *Vegfa* | QT00160769 |
| *Vegfb* | QT00159863 |
